# c-Abl Mediated Tyrosine Phosphorylation of Aha1 Activates Its Co-chaperone Function in Cancer Cells

**DOI:** 10.1016/j.celrep.2015.07.004

**Published:** 2015-07-30

**Authors:** Diana M. Dunn, Mark R. Woodford, Andrew W. Truman, Sandra M. Jensen, Jacqualyn Schulman, Tiffany Caza, Taylor C. Remillard, David Loiselle, Donald Wolfgeher, Brian S.J. Blagg, Lucas Franco, Timothy A. Haystead, Soumya Daturpalli, Matthias P. Mayer, Jane B. Trepel, Rhodri M.L. Morgan, Chrisostomos Prodromou, Stephen J. Kron, Barry Panaretou, William G. Stetler-Stevenson, Steve K. Landas, Len Neckers, Gennady Bratslavsky, Dimitra Bourboulia, Mehdi Mollapour

**Affiliations:** 1 Department of Urology, SUNY Upstate Medical University, 750 East Adams Street, Syracuse, NY 13210, USA; 2 Department of Biochemistry and Molecular Biology, SUNY Upstate Medical University, 750 East Adams Street, Syracuse, NY 13210, USA; 3 Cancer Research Institute, SUNY Upstate Medical University, 750 East Adams Street, Syracuse, NY 13210, USA; 4 Department of Biological Sciences, University of North Carolina Charlotte, Charlotte, NC 28223, USA; 5 Department of Molecular Genetics and Cell Biology, University of Chicago, Chicago, IL 60637, USA; 6 Radiation Oncology Branch, Center for Cancer Research, National Cancer Institute, 9000 Rockville Pike, Bethesda, MD 20892, USA; 7 Department of Pathology, SUNY Upstate Medical University, 750 East Adams Street, Syracuse, NY 13210, USA; 8 Department of Pharmacology and Cancer Biology, Duke University Medical Center, Durham, NC 27710, USA; 9 Department of Medicinal Chemistry, University of Kansas, 1251 Wescoe Hall Drive, Lawrence, KS 66045, USA; 10 Zentrum für Molekulare Biologie der Universitat Heidelberg, DKFZ-ZMBH-Alliance, Heidelberg 69120, Germany; 11 Developmental Therapeutics Branch, National Cancer Institute, 9000 Rockville Pike, Bethesda, MD 20892, USA; 12 Genome Damage and Stability Centre, University of Sussex, Brighton BN1 9RQ, UK; 13 Institute of Pharmaceutical Science, Kings College London, London SE1 9NH, UK; 14 Urologic Oncology Branch, Center for Cancer Research, National Cancer Institute, 9000 Rockville Pike, Bethesda, MD 20892, USA

## Abstract

The ability of Heat Shock Protein 90 (Hsp90) to hydrolyze ATP is essential for its chaperone function. The co-chaperone Aha1 stimulates Hsp90 ATPase activity, tailoring the chaperone function to specific “client” proteins. The intracellular signaling mechanisms directly regulating Aha1 association with Hsp90 remain unknown. Here, we show that c-Abl kinase phosphorylates Y223 in human Aha1 (hAha1), promoting its interaction with Hsp90. This, consequently, results in an increased Hsp90 ATPase activity, enhances Hsp90 interaction with kinase clients, and compromises the chaperoning of non-kinase clients such as glucocorticoid receptor and CFTR. Suggesting a regulatory paradigm, we also find that Y223 phosphorylation leads to ubiquitination and degradation of hAha1 in the proteasome. Finally, pharmacologic inhibition of c-Abl prevents hAha1 interaction with Hsp90, thereby hypersensitizing cancer cells to Hsp90 inhibitors both in vitro and ex vivo.

## Graphical abstract

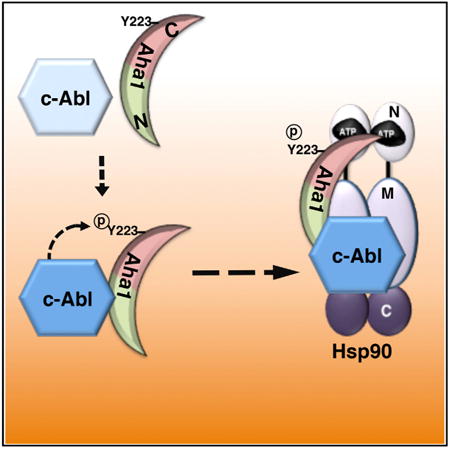

## Introduction

The essential eukaryotic molecular chaperone Heat Shock Protein 90 (Hsp90) is involved in folding and stability of target proteins, also referred to as “clients” (Röhl et al., 2013; Taipale et al., 2010). Hsp90 has approximately 200 clients (listed at http://www.picard.ch/downloads/Hsp90interactors.pdf). They are broadly classified as kinase clients, such as ErbB2, c-Met, and CDK4 and non-kinase clients including heat shock factor, steroid hormone receptors, and cystic fibrosis transmembrane conductance regulator (CFTR). The majority of the kinase clients are involved in oncogenesis, therefore Hsp90 is recognized as a facilitator of “oncogene addiction” (Neckers and Workman, 2012).

The Hsp90 structure consists of homodimer molecules with N-, middle, and C-domains. ATP binding to the N-domain and its subsequent hydrolysis are linked to Hsp90 chaperone function (Obermann et al., 1998; Panaretou et al., 1998). Nucleotide binding and Hsp90 ATPase activity confer different conformational states that allow clients to bind and dissociate from Hsp90 (Hessling et al., 2009; Mickler et al., 2009). The chaperone activity of Hsp90 is tightly regulated by co-chaperones and post-translational modifications (PTMs) (Cox and Johnson, 2011; Walton-Diaz et al., 2013). Co-chaperones are groups of proteins that interact with distinct conformations of Hsp90, regulating chaperone function by either accelerating or decelerating the ATPase activity or simply acting as scaffolds between Hsp90 and its clients. Our work and studies by other groups have shown that PTMs of Hsp90 can impact its interaction with co-chaperones. The evolutionarily conserved co-chaperone Aha1 is the activator of the Hsp90 ATPase activity (Panaretou et al., 1998). It is also the most common co-chaperone whose interaction is affected by phosphorylation, acetylation, and SUMOylation of Hsp90 (Mollapour et al., 2011, 2014; Xu et al., 2012).

A major gap in our knowledge is how intracellular signals to the co-chaperone Aha1 dictate its interaction with Hsp90. Our study demonstrates that c-Abl tyrosine kinase phosphorylates a single tyrosine residue, Y223, in human Aha1 (hAha1). This, in turn, appears to promote its association with human Hsp90α (hHsp90α) and modify chaperoning of kinase clients, heat shock factor, glucocorticoid receptor (GR), and CFTR. Tyrosine phosphorylation of hAha1 is also a pre-requisite for its ubiquitination and degradation in the proteasome.

Hsp90 chaperone function can be inhibited by small molecules that bind to the N-domain ATP-binding pocket, precluding ATP binding and hydrolysis. There are 16 different Hsp90 inhibitors that are currently undergoing clinical evaluation in cancer patients (Neckers and Workman, 2012). Co-chaperones and PTMs can affect the efficacy of Hsp90 inhibitors (Walton-Diaz et al., 2013). Here, we report that the pharmacologic inhibition of c-Abl prevents hAha1 interaction with hHsp90α and hypersensitizes renal cell carcinoma (RCC) to Hsp90 inhibitors in vitro and ex vivo.

## Results

### c-Abl Phosphorylates Y223 in the Co-chaperone Aha1

**Figure 1:**
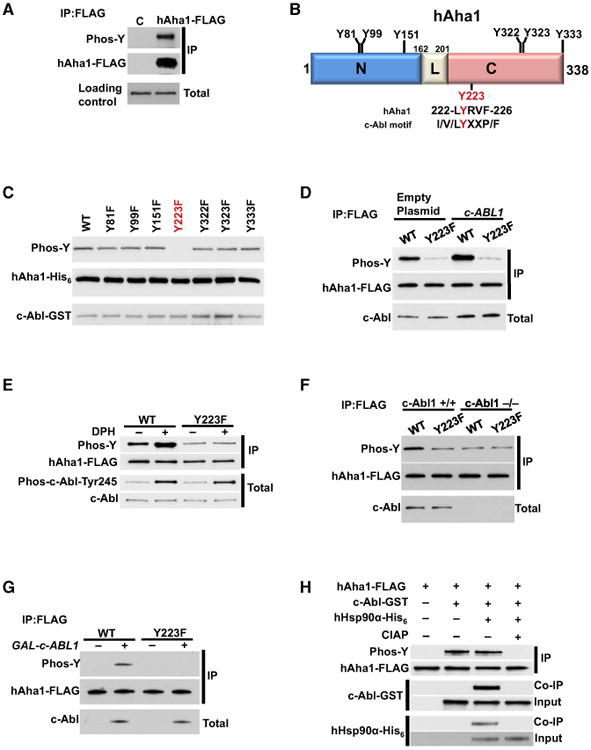
c-Abl Mediated Tyrosine Phosphorylation of hAha1 (A) HEK293 cells were transiently transfected with empty plasmid (C) or hAha1-FLAG construct. The hAha1-FLAG was immunoprecipitated (IP) and tyrosine phosphorylation was detected by immunoblotting with pan anti-phospho-tyrosine antibody (4G10). (B) Schematic representation of hAha1 showing all tyrosine residues and also the putative c-Abl targeted tyrosine consensus motif. The amino acid residues 162-201 correspond to linker (L) region. (C) hAha1 tyrosine residues were mutated individually to phenylalanine (F), expressed, and purified from bacteria. The active c-Abl-GST was used to phosphorylate hAha1 wild-type (WT) and its non-phosphorylatable mutants in vitro. The tyrosine phosphorylation was detected by immunoblotting with pan anti-phospho-tyrosine antibody (4G10). (D) HEK293 cells were transiently transfected with c-Abl and hAha1-FLAG (WT) or the non-phosphorylatable Y223F mutant. The tyrosine phosphorylation of IP hAha1-FLAG was detected by immunoblotting with pan anti-phospho-tyrosine antibody (4G10). (E) hAha1-FLAG (WT) or the non-phosphorylatable Y223F mutant constructs were used to transiently transfect HEK293 cells for 24 hr. The cells were then treated with 20 μM DPH for 6 hr prior to lysis. The tyrosine phosphorylation of IP hAha1-FLAG was detected by immunoblotting with pan anti-phospho-tyrosine antibody (4G10). (F) c-Abl deficient (c-Abl−/−) MEF cell line and the WT MEF cell line (c-Abl+/+) were transiently transfected with hAha1-FLAG (WT) or the non-phosphorylatable Y223F mutant. The IP hAha1-FLAG was immunoblotted and tyrosine phosphorylation was detected with pan anti-phospho-tyrosine antibody (4G10). (G) WT yeast cells co-expressing *GAL1-cABL1* and *hAHA1*-FLAG or Y223F mutant under *yAHA1* native promoter were grown on either raffinose (–) or galactose (+). The tyrosine phosphorylation of hAha1 was assessed by IP and immunoblotting. (H) In vitro phosphorylation of hAha1-Y223 by a purified and active c-Abl. The hAha1-FLAG or hAha1-Y223F-FLAG were bound to FLAG-beads and phosphorylated by active c-Abl-GST. The reaction was also carried out with the addition of hHsp90α-His_6_. The dephosphorylation of hAha1-Y223 was performed by addition of CIAP.

Hsp90 and the majority of its co-chaperones are phospho-proteins (Walton-Diaz et al., 2013). To determine whether Aha1 is subject to tyrosine phosphorylation, hAha1-FLAG was transiently expressed in HEK293 cells and by using a pan-anti-phospho-tyrosine antibody (4G10), we readily detected the tyrosine phosphorylation of hAha1 (Figure 1A). hAha1 has seven tyrosine residues (Figure 1B), which were individually mutated to non-phosphorylatable phenylalanine and transiently expressed in HEK293 cells. Individual mutation of Y81, Y99, Y223, and Y333 to phenylalanine significantly reduced the tyrosine phosphorylation of hAha1 (Figure S1A). We identified Y223 within the c-Abl recognition motif I/V/L-YXXP/F (Ubersax and Ferrell, 2007) (Figure 1B). Therefore, we bacterially expressed and purified N-terminally His_6_-tagged hAha1, as well as the seven individual non-phosphorylatable hAha1 mutants. These purified proteins were bound to Ni-NTA agarose and used as substrates in an in vitro kinase assay, which included pure and active c-Abl-glutatione S-transferase (GST). Under these conditions, c-Abl-GST efficiently phosphorylated hAha1 and individual non-phosphorylatable tyrosine mutants except for the Y223F (Figure 1C). These results provide strong evidence that c-Abl directly phosphorylates Y223-hAha1, and this is the only tyrosine residue in hAha1 that is targeted by c-Abl (Figure 1C).

Further evidence for this process, in vivo, was obtained by transiently co-transfecting HEK293 cells with c-Abl plasmid and either with FLAG-tagged hAha1 or non-phosphorylatable Y223F mutant. Overexpression of c-Abl increased the tyrosine phosphorylation of hAha1. However, this increase in tyrosine phosphorylation is abolished with hAha1-Y223F mutant (Figure 1D). We also performed similar experiments with 5-(1,3-diaryl-1H-pyrazol-4-yl)hydantoin, 5-[3-(4-fluorophenyl)-1-phenyl-1H-pyrazol-4-yl]-2,4-imidazolidinedione (DPH), which binds to the myristoyl binding site of c-Abl and leads to activation of its kinase activity (Yang et al., 2011). HEK293 cells transiently expressing wild-type hAha1-FLAG or Y223F mutant were treated with 20 μM DPH showed an increase in tyrosine phosphorylation of hAha1, but not of the Y223F mutant (Figure 1E).

We further confirmed this data by transiently expressing FLAG-hAha1 and Y223F mutant in a c-Abl deficient (c-Abl−/−) murine embryo fibroblasts (MEF) cell line and the wild-type MEF cell line (c-Abl+/+) (Figure 1F). Tyrosine phosphorylation of hAha1 was significantly reduced in c-Abl deficient MEF cells (Figure 1F). This reduction was at the same level of Y223F mutant expressed either in the c-Abl+/+ or c-Abl−/− MEF cells (Figure 1F).

With the exception of the Wee1 tyrosine kinase, the yeast *Saccharomyces cerevisiae* does not have a bona fide tyrosine kinase offering a null background for the expression of mammalian tyrosine kinase c-Abl. We expressed FLAG-tagged hAha1 and the Y223F mutant in wild-type yeast strain W303; expression of these alleles was under the control of the native promoter of yeast *AHA1* (*yAHA1*). We also co-expressed full-length c-Abl, controlled by the galactose inducible promoter of *GAL1*. Tyrosine phosphorylation of hAha1 was not observed in wild-type yeast (Figure 1G). However, after inducing *c-ABL1* expression by growing the cells on galactose media, we detected tyrosine phosphorylation of wild-type hAha1. The non-phosphorylatable Y223F mutant remained unmodified (Figure 1G). Taken together, our data provide strong evidence that c-Abl targets and phosphorylates hAha1-Y223.

Subsequently, we investigated whether phosphorylation of the hAha1-Y223 occurred before or after binding to hHsp90. The presence of endogenous hHsp90 and lack of efficient knockdown of hHsp90 precluded addressing this issue in mammalian cell lines. Therefore, we utilized our in vitro kinase assay to explore the dynamics of hAha1-Y223 phosphorylation. Bacterially expressed and purified FLAG-hAha1 was immobilized on anti-FLAG M2 affinity gel. As shown earlier, (Figure 1C), purified and active c-Abl-GST phosphorylates hAha1 (Figure 1H). This was detected using a pan-anti-phosphotyrosine antibody. Immunoprecipitation of FLAG-hAha1 did not co-immunoprecipitate c-Abl-GST, suggesting that the interaction between hAha1 and c-Abl is transient (Figure 1H). We carried out a similar in vitro kinase assay, modified with the addition of hHsp90α, initially, followed by c-Abl. This promoted a hAha1-c-Abl-hHsp90α complex formation, but did not increase the tyrosine phosphorylation of hAha1 (Figure 1H). Addition of calf-intestinal alkaline phosphatase (CIAP) caused dephosphorylation of hAha1 and de-stabilized the hAha1-c-Abl-Hsp90α complex (Figure 1H, last lane). Also, c-Abl does not phosphorylate hHsp90α (Figure S1B). Taken together, our data suggest that c-Abl has the ability to phosphorylate hAha1-Y223 even in the absence of hHsp90α. This fosters formation of a hAha1-c-Abl-hHsp90α complex.

### Y223 Phosphorylation Leads to hAha1 Ubiquitination and Degradation in Proteasomes

**Figure 2:**
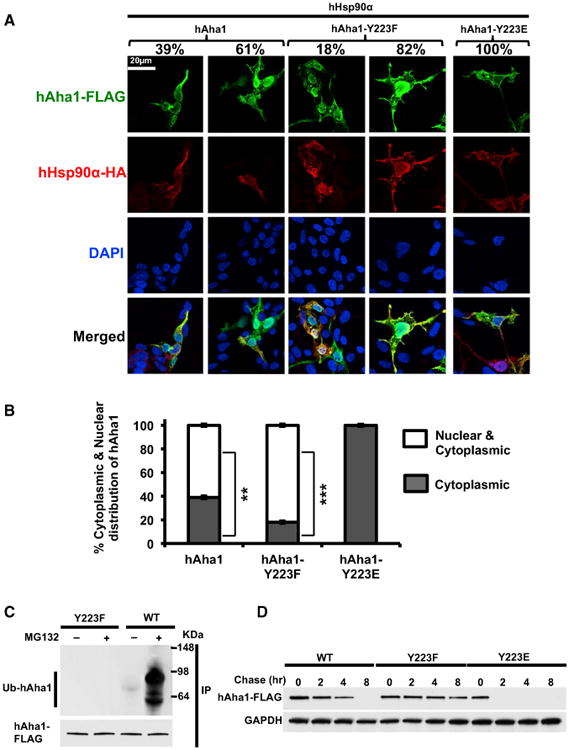
Y223 Phosphorylation Leads to Ubiquitination and Degradation of hAha1 in Proteasomes (A) HEK293 cells were co-transfected with hHsp90α-HA and either empty plasmid pcDNA3, hAha1-FLAG, Y223F, or Y223E mutants. The cells were analyzed by immunofluorescence microscopy. (B) Cytoplasmic and nuclear distribution of hAha1 and Y223F and Y223E mutants in (A) are presented as average percentage. All the data represent mean ± SD (**p < 0.005, ***p < 0.0005). (C) HEK293 cells were transiently transfected with hAha1-FLAG and Y223F mutant for 24 hr and then treated with 5 μM MG132 for 6 hr. The hAha1-FLAG and Y223F mutants were immunoprecipitated (IP) and ubiquitination was assessed by immunoblotting with anti-ubiquitin antibody. The IP samples were salt-stripped (0.5 M NaCl) prior to immunoblotting. (D) hAha1-FLAG (WT), Y223F, or Y223E mutants were transiently transfected in HEK293 cells for 24 hr. The cells were then treated with CHX (100 μg/ml) and harvested at the indicated time points. The hAha1 was visualized by immunoblotting.

Our data suggest a model where tyrosine phosphorylation of hAha1 promotes interaction, while a possible dephosphorylation disrupts interaction with hHsp90. However, we sought to examine whether phosphorylation of hAha1 serves any additional functions. First, we characterized the intracellular distribution of hAha1 as the result of phosphorylation of Y223. HEK293 cells were transiently co-transfected with hHsp90α-HA, wild-type hAha1-FLAG, and non-phosphorylatable Y223F or phosphomimetic Y223E mutants. We performed immunofluorescence microscopy, staining the cells with anti-FLAG antibody and co-staining with anti-HA antibody and DAPI. There were 82% of cells expressing the hAha1-Y223F mutant that showed nuclear-cytoplasmic localization and 18% with cytoplasmic localization, whereas 100% of cells expressing the Y223E mutant displayed cytoplasmic localization (Figures 2A, 2B, and S2; Table S1).

Protein phosphorylation often serves as a signal prompting ubiquitination and subsequent proteasome-mediated degradation of the substrate. Supporting this possibility, HEK293 cells transiently expressing the wild-type hAha1-FLAG or non-phosphorylatable hAha1-Y223F-FLAG mutant were treated with 5 μM proteasome inhibitor, MG132, for 6 hr (Figure 2C). hAha1-FLAG proteins were immunoprecipitated with anti-FLAG M2 affinity gel and then salt-stripped with 0.5 M NaCl prior to analysis by immunoblotting (Figure 2C). Ubiquitination of hAha1 was significantly decreased in hAha1-Y223F samples treated with MG132 (Figure 2C). This finding suggests that phosphorylation of Y223 is a prerequisite for ubiquitination and degradation of hAha1 in the proteasome. It is noteworthy that salt-stripping of our samples prevented the interaction of proteins with hAha1, and the efficacy of the salt-stripping procedure was confirmed by checking for the co-precipitation of hHsp90 (data not shown). Therefore, only ubiquitinated hAha1 is visualized in Figure 2B, as opposed to ubiquitinated proteins in complex with hAha1.

Finally, the rate of hAha1 protein degradation was determined by cycloheximide (CHX) chase analysis. The non-phosphorylatable hAha1-Y223F mutant was markedly more stable compared to the wild-type hAha1 (Figure 2D). Conversely, the phosphomimetic Y223E mutant was highly unstable supporting a model where c-Abl mediated phosphorylation of Y223 leads to hAha1 ubiquitination and degradation in the proteasome.

### Impact of hAha1 Phospho-Y223 Mutants on hHsp90 Chaperone Activity

Aha1 interacts with a diverse range of proteins (Sun et al., 2015). To better understand the effect of phosphorylation on the global interactions of hAha1, we applied a quantitative liquid chromatography-tandem mass spectrometry (LC–MS/MS) proteomics approach, comparing the interactomes of FLAG-hAha1-Y223E to FLAG-hAha1-Y223F (Figure S3A; Table S2). The mass spectrometry proteomics data have been deposited to the ProteomeXchange Consortium via the PRIDE partner repository with the dataset identifier PXD001737. We identified 99 candidate partners of hAha1, 84% (83/99) of which demonstrated preferential binding to the phosphomimetic isoform of hAha1 (Figures S3B and S3C; Table S2). Significant enrichment of several Gene Ontology (GO) terms was observed, including metabolism, ribosomal components, and transcription/translation (Figure S3B).

**Figure 3:**
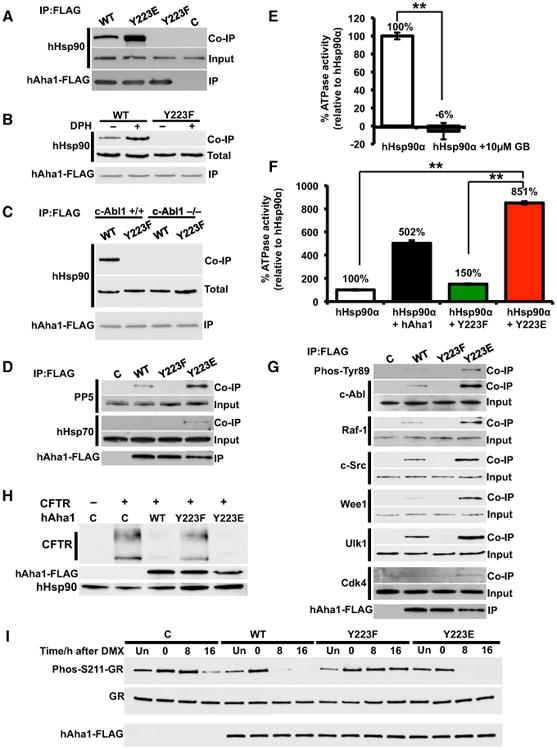
Tyrosine Phosphorylation of hAha1 Is Essential for Binding to Hsp90 (A) HEK293 cells were transiently transfected with empty plasmid (C), WT hAha1-FLAG (WT), non-phosphorylatable Y223F, or phosphomimetic Y223E mutants. The hAha1-FLAG protein was immunoprecipitated (IP) and co-immunoprecipitating (co-IP) of hHsp90 (including α and β isoforms) was examined by immunoblotting. (B) HEK293 cells transiently expressing hAha1-FLAG (WT) or the non-phosphorylatable Y223F mutant were treated with 20 μM DPH for 6 hr prior to lysis. The hAha1-FLAG proteins were immunoprecipitated (IP) and co-immunoprecipitating (co-IP) hHsp90 was detected by immunoblotting. (C) The c-Abl+/+ and c-Abl deficient c-Abl−/− MEF cells were transiently transfected with hAha1-FLAG (WT) or the non-phosphorylatable Y223F mutant. The immunoprecipitation of hAha1-FLAG proteins and co-immunoprecipitating (co-IP) of hHsp90 were detected by immunoblotting. (D) Co-immunoprecipitating (co-IP) of PP5 and Hsp70 in (A) were examined by immunoblotting. (E) In vitro ATPase activity of hHsp90α-HA isolated from PC3 cells. The reaction was also performed in the presence of 10 μM ganetespib. All the data represent mean ± SD (**p < 0.005). (F) The ATPase activity was stimulated by hAha1-FLAG (WT) or the phospho mutants Y223E and Y223F isolated from HEK293 cells. All the data represent mean ± SD (**p < 0.005). (G) Indicated hAha1-FLAG constructs were transfected in HEK293 cells. The hAha1 was immunoprecipitated (IP) with anti-FLAG agarose; co-immunoprecipitating (co-IP) c-Abl and phospho-Tyr89, Raf-1, c-Src, Wee1, Ulk1, and Cdk4 proteins were detected by immunoblotting. (H) HEK293 cells were co-transfected with CFTR and hAha1-FLAG (WT) and Y223F and Y223E mutants. After 24 hr, CFTR, hAha1-FLAG, and hHsp90 were detected by immunobloting. (I) HEK293 cells transiently transfected with hAha1-FLAG, Y223F, and Y223E for 24 hr and then treated with 10 μM dexamethasone for 1 hr (time 0). The cells were then washed and incubated in dexamethasone free media. The GR activity was monitored by western blot analysis using anti-phospho-Ser211-GR and total GR antibodies.

Interestingly, binding to major chaperones, including human Hsp70 (hHsp70) and hHsp90 was significantly increased upon hAha1 phosphorylation. hAha1 is known to interact and activate hHsp90 ATPase activity (Panaretou et al., 2002). Phosphorylation of hHsp90 can affect its binding to hAha1 (Mollapour et al., 2011; Soroka et al., 2012; Xu et al., 2012), therefore, we queried whether phosphorylation of hAha1 impacts binding to hHsp90. FLAG-tagged wild-type hAha1, non-phosphorylatable Y223F, and phosphomimetic Y223E mutants were transiently expressed in HEK293 cells. hAha1 proteins were isolated with anti-FLAG M2 affinity gel and interaction with hHsp90 and co-chaperones was examined by western blot analysis. In agreement with our proteomic study, the non-phosphorylatable hAha1-Y223F association with Hsp90 was completely abrogated (Figure 3A). Conversely, the interaction of hAha1-Y223E mutant with hHsp90α was significantly stronger than the wild-type hAha1 (Figure 3A). In order to directly demonstrate that the phosphorylation of hAha1 promotes its interaction with hHsp90, we treated HEK293 cells transiently expressing wild-type hAha1-FLAG or Y223F mutant with 20 μM DPH and then isolated these proteins by anti-FLAG M2 affinity gel. The hHsp90 was co-immunoprecipitated with hAha1-FLAG, and this interaction was enhanced in DPH treated cells (Figure 3B). However, we did observe hAha1-Y223F association with hHsp90 even in DPH treated cells (Figure 3B). Similar results were also obtained from c-Abl−/− MEF cells (Figure 3C), therefore providing convincing evidence that c-Abl mediated phosphorylation of hAha1 promotes its interaction with hHsp90. We further validated our proteomic data by observing hHsp70 interaction with the phosphomimetic hAha1 mutant Y223E, presumably mediated by hHsp90 (Figure 3D). We have previously shown that the PP5 co-chaperone is also found in complexes containing hAha1 and Hsp90 (Xu et al., 2012). The phosphomimetic hAha1-Y223E mutant moderately enhanced the amount of PP5 found in these complexes, in comparison to the amount of PP5 that was co-immunprecipitated with the wild-type hAha1 (Figure 3D). In contrast, PP5 is absent in complexes that contain the hAha1-Y223F mutant (Figure 3D). It is noteworthy that neither the wild-type hAha1 nor the phospho mutants were able to form a complex with the other co-chaperones (HOP, p23, CHIP, and Cdc37), (Figure S3C).

ATPase activity of hHsp90 is essential for its chaperone function and hAha1 is a potent stimulator of this activity (Panaretou et al., 2002). We transiently expressed hHsp90α-HA in the prostate cancer PC3 cell line, wild-type hAha1-FLAG, and the Y223 phospho mutants in HEK293 cells. Proteins were immunoprecipitated, salt stripped, and competed off the HA or FLAG affinity beads with the relevant peptides (Figure S4A). The quality of the isolated proteins was examined by Coomassie staining of the SDS-PAGE (Figure S4B). These purified proteins were quantified and used at a ratio of 1:2, hHsp90α:hAha1 in the PiPer Phosphate Assay Kit (Thermo Fisher Scientific), (Experimental Procedures) in the presence of ATP as substrate as previously described (Kamal et al., 2003). We measured the ATPase activity of isolated hHsp90α in vitro as previously described (Kamal et al., 2003) (Figures 3E, S4C, and S4D). Addition of 10 μM ganetespib inhibited ATPase activity (Figures 3E and S4D).

Addition of wild-type hAha1, comprised of phospho- and non-phosphorylated Y223, stimulated ATPase activity of the hHsp90α 5-fold higher than that of hHsp90α, alone, while hAha1-Y223F caused minimal increase in ATPase activity (1.5-fold increase) (Figures 3F and S4E). In contrast, the phosphomimetic hAha1-Y223E mutant stimulated hHsp90α ATPase by 8.5-fold (Figures 3F and S4E). ATP turnover was expressed as mmol P_i_ per mol per minute for hHsp90α alone and in the presence of hAha1 (wild-type and Y223 mutants), (0.84 = hHsp90α), (4.22 = hHsp90α + wild-type hAha1), (1.26 = hHsp90α + hAha1-Y223F), and (7.15 = hHsp90α and hAha1-Y223E), (Figure S4E). These data are comparable to previously reported ATPase turnover rates of hHsp90α (Kamal et al., 2003). Also, overexpression of wild-type hAha1 or the phospho mutants did not affect the Hsp90 binding to ATP-agarose (Figure S4F). It is noteworthy that isolated hAha1 and the mutant proteins had no contaminating ATPase activity (Figure S4D).

We previously demonstrated that hAha1 co-exists in an hHsp90-kinase client complex (Xu et al., 2012). Therefore, we assessed the impact of Y223F and Y223E mutations on hAha1 interaction with kinase clients. We transiently expressed FLAG-tagged wild-type hAha1 and the Y223 phospho mutants in HEK293 cells. Following immunoprecipitation with anti-FLAG M2 affinity gel, we observed hAha1 in complex with the endogenous active (phospho-S89) c-Abl, Raf-1, c-Src, Wee1, and Ulk1 kinases, but not Cdk4 (Figure 3G). These kinases formed a stronger interaction with the phosphomimetic Y223E mutant than did the wild-type hAha1 (Figure 3G). Conversely, association of the non-phosphorylatable Y223F mutant with c-Abl, Raf-1, c-Src, Wee1, and Ulk1 was completely abolished (Figure 3G). Overexpression of hAha1 compromises the folding of CFTR (Koulov et al., 2010; Wang et al., 2006). To examine the effects of Y223 phospho mutants on Hsp90 chaperone function, we assessed their impact on steady-state expression of CFTR protein in mammalian cells. HEK293 cells were transiently co-transfected with CFTR and FLAG-hAha1, or Y223F or Y223E mutants. Empty plasmid pcDNA3 (C) was used as a negative control. Western blot analysis of these samples using anti-CFTR antibody detected a doublet (Figure 3H), with the upper band representing the mature Golgi-processed glycoform of CFTR found at the cell surface and the lower band an immature core-glycosylated protein (Figure 3H). Overexpression of the non-phosphorylatable Y223F mutant did not reduce the stability of CFTR (Figure 3H), confirming its inability to bind and activate hHsp90. In contrast, overexpression of the phosphomimetic hAha1-Y223E mutant displayed a significant reduction of CFTR protein (Figure 3H). The enhanced interaction of Y223E-hAha1 mutant with hHsp90 increases chaperone activity and, therefore, reduces the expression of CFTR. Finally, we transiently expressed hAha1-FLAG and Y223 phosphomutants in HEK293 cells and then stimulated the cells with 10 μM dexamethasone for 1 hr, representing time 0. Cells were then washed and incubated in dexamethasone free media. GR activity was monitored by western blot analysis using anti-phospho-Ser211-GR antibody. Overexpression of the non-phospho-hAha1-Y223F mutant maintained the GR activity even 16 hr post dexamethasone treatment (Figure 3I). However, overexpression of the phosphomimetic hAha1-Y223E caused a rapid reduction in GR activity (Figure 3I).

These data confirm the hypothesis that phosphorylation of hAha1-Y223 increases binding to Hsp90 and compromises the chaperoning and the activity of the clients that are “difficult”to fold.

### Phosphorylation of hAha1-Y223 Affects Chaperoning of the Clients

**Figure 4:**
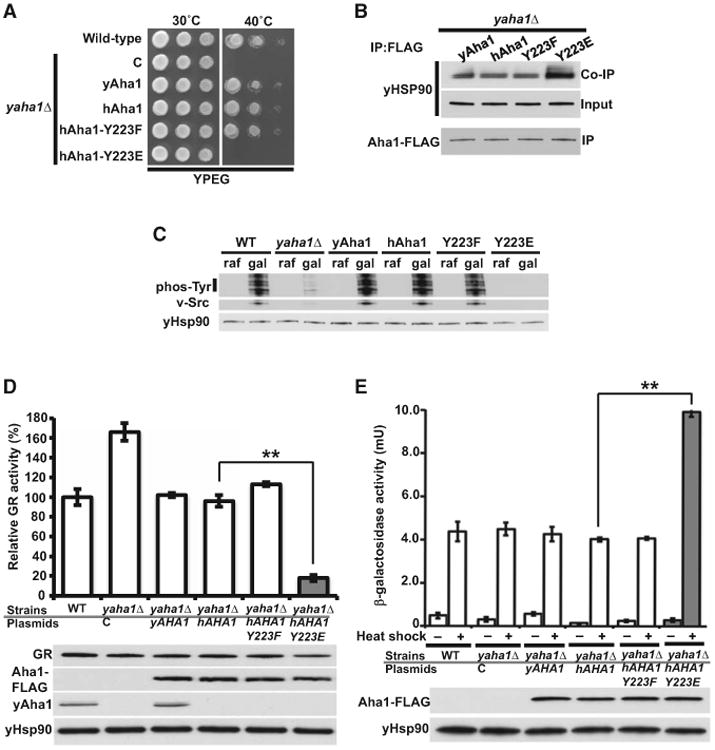
Phosphorylation of hAha1-Y233 Impacts the Chaperoning of Hsp90 Clients (A) *yAha1* deleted cells (*yaha1*Δ) expressing empty-plasmid pRS314 (C), yAha1, hAha1, hAha1-Y223F, or hAha1-Y223E mutants were grown on YPEG (respiratory) liquid media at 28°C for 24 hr and 1:10 dilution series were spotted on YPEG agar. Plates were incubated at either 30°C or 40°C for 5 days. (B) Yeast cells in (A) expressing indicated Aha1-FLAG under *yAHA1* native promoter were used to immunoprecipitate (IP) Aha1 with anti-FLAG agarose. The co-immunoprecipitation (co-IP) of yHsp90 was analyzed by immunoblotting. (C) Indicated yeast cells containing *v-SRC* under *GAL1* promoter were grown on raffinose (–) or galactose (+) media. v-Src and total phosphotyrosine were analyzed by immunoblotting. (D) GR-*lacZ* activity was assessed in the indicated yeast strains. The data are expressed as a percentage of the activity observed in WT cells. The mean ± SD from values obtained in three independent experiments with **p < 0.005 are presented. The levels of yAha1, hAha1-FLAG, yHsp90, and GR were analyzed by immunoblotting. (E) Yeast strains in (A) with HSE-*lacZ* were heat shocked (40 min at 39°C). The heat shock response activity was measured in three independent experiments. The hAha1-FLAG and yHsp90 protein levels were assessed by immunoblotting. All the data represent mean ± SD (**p <0.005).

The budding yeast *S. cerevisiae* was used to further investigate the impact of hAha1 Y223 phosphorylation on Hsp90 chaperone function. Aha1 is an evolutionarily conserved co-chaperone, and it has been previously shown that deletion of *yAHA1* renders cells temperature-sensitive on glucose (YPED) and respiratory growth media (YPEG) (Panaretou et al., 1998). Since we did not observe tyrosine phosphorylation of hAha1 in yeast (data not shown), we hypothesized that yAha1 acts through a different mechanism to initiate its interaction with yHsp90. Therefore, the same effect should be displayed when either hAha1 or the non-phosphorylatable hAha1-Y223F mutant is expressed in yeast. This turned out to be the case, as overexpression of *hAHA1* and the Y223F mutant, under control of the y*AHA1* native promoter on a centromeric (single copy) plasmid, reverted the temperature sensitivity phenotype of the *yaha1* knockout cells on both YPED and YPEG media (Figure 4A). Furthermore, both forms of hAha1 bound to yHsp90 similarly to wild-type yAha1 (Figure 4B). Expression of the phosphomimetic Y223E mutant did not revert the temperature-sensitivity of *yaha1*Δ cells on YPEG (Figure 4A) and, compared to the wild-type yAha1, its association to yHsp90 was enhanced (Figure 4B). These data indicate that expression of Y223E in yeast has a dominant-negative effect on initiating its binding with Hsp90. These results are also concordant with our observations in mammalian cells.

We employed these yeast strains to examine the effects of the Y223E mutant on chaperoning of kinase and non-kinase clients. The tyrosine kinase, v-Src, is an established Hsp90 client, and its expression in yeast causes cell death (Nathan and Lindquist, 1995). v-Src expression and upregulation of protein tyrosine phosphorylation as the result of v-Src activity was significantly reduced in the *yaha1*Δ yeast (Figure 4C), implying compromised Hsp90 chaperone function. Expression of *yAHA1, hAHA1*, or the non-phosphorylatable Y223F mutant restored the defect in yHsp90 function. In contrast, v-Src expression and tyrosine phosphorylation of the protein lysate was undetectable in the hAha1-Y223E mutant (Figure 4C).

GR is another well-characterized Hsp90 non-kinase client that provides a sensitive assay for Hsp90 function in yeast (Garabedian and Yamamoto, 1992). To assess the impact of Y223 mutation on GR activity, we transformed the above yeast cells with a GR expression plasmid also carrying a glucocorticoid-regulated *LacZ* reporter gene. Deletion of *yAHA1* increased the GR activity by 66% (Figure 4D). Expression of yeast, hAha1, or Y223F mutant reduced the GR activity to the same levels as in the wild-type cell (Figure 4D). Yeast cells expressing the phosphomimetic Y223E mutant had only 18% GR activity relative to wild-type cells (Figure 4D).

We also tested the effect of hAha1 phosphorylation on *HSF1* transcriptional activity. Previous work showed that Hsf1 is an Hsp90 client, and their interaction suppresses Hsf1 activity (Hjorth-Sørensen et al., 2001). Compromised Hsp90 chaperone function leads to induction of Hsf1 activity in yeast even in the absence of heat shock (Hjorth-Sørensen et al., 2001). Although deletion of *yAHA1* in yeast did not affect the heat shock response, expression of the phosphomimetic Y223E mutant led to a 2.5-fold increase in the heat shock response upon heat shock stress compared to the wild-type hAha1 cells (Figure 4E). Taken together, these data suggest that expression of the Y223E affects the chaperoning of kinase and non-kinase clients such as v-Src, GR, and Hsf1.

### Pharmacological Inhibition of c-Abl Sensitizes Cancer Cells to Hsp90 Inhibitors

**Figure 5:**
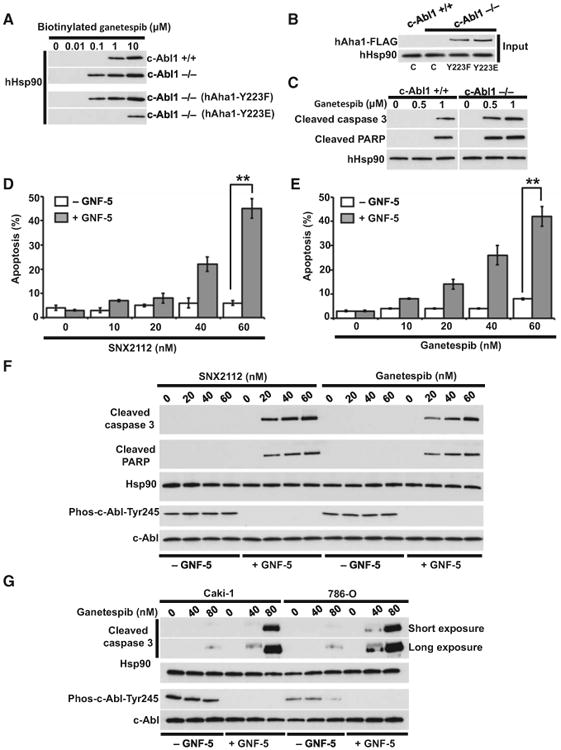
c-Abl Inhibition Hypersensitizes Prostate Cancer and RCC Cell Lines to Hsp90 Inhibitors (A) Lysates from WT (c-Abl+/+) and c-Abl deficient (c-Abl−/−) MEF cell lines also carrying indicated hAha1 phospho mutant plasmids were incubated with indicated amounts of biotinylated ganetespib, followed by streptavidin agarose beads. The hHsp90α/β was detected by immunoblotting with anti-hHsp90 monoclonal antibody (16F1). (B) Total lysate from samples in (A) were tested for hHsp90 by immunoblotting with anti-hHsp90 (16F1) and anti-FLAG antibodies. (C) WT (c-Abl+/+) and c-Abl deficient (c-Abl−/−) MEF cell lines were treated with indicated amounts of ganetespib for 6 hr. The cleaved PARP and cleaved caspase-3 were detected by immunoblotting. The hHsp90 was used as a loading control. (D and E) Prostate cancer PC3 cells were treated with 5 μM c-Abl inhibitor GNF-5 for 24 hr. The cells were then treated with indicated concentrations of Hsp90 inhibitors SNX2112 and (E) ganetespib for an additional 24 hr. Apoptosis was detected by FACS analysis. The errors bars in (D) and (E) represent the SD of three independent experiments (**p < 0.005). (F) PC3 cells were treated with 5 μM c-Abl inhibitor GNF-5 for 24 hr and then with indicated concentrations of SNX2112 and ganetespib for an additional 24 hr. Hsp90, c-Abl, active c-Abl (phospho-Y245), and apoptosis indicator cleaved caspase-3 and cleaved PARP were detected by immunoblotting. (G) RCC Caki-1 and 786-O cell lines were treated with 5 μM c-Abl inhibitor GNF-5 for 24 hr and then with indicated concentrations of SNX2112 and ganetespib for an additional 24 hr. Hsp90, c-Abl, active c-Abl (phospho-Y245), and apoptosis indicators cleaved caspase-3 and cleaved PARP were detected by immunoblotting.

Previous work has shown that knock down of hAHA1 could sensitize cancer cells to Hsp90 inhibitors (Holmes et al., 2008). Lack of phosphorylation of Y223 prevents hAha1 from binding to hHsp90 (Figure 3A), and this should phenocopy the effect of hAha1 knockdown. It follows that any strategy that limits hAha1 association with hHsp90 should have the same effect as depletion of hAha1, namely sensitization of cells to Hsp90 inhibitors. Accordingly, given that c-Abl phosphorylates Y223, we hypothesized that absence or pharmacological inhibition of c-Abl would sensitize cancer cells to Hsp90 inhibitors. We first explored c-Abl−/− and c-Abl+/+ MEF cell lines using the bio-tinylated Hsp90 inhibitor, ganetespib, and streptavidin beads to affinity purify hHsp90 protein from cell lysates. We observed that lack of c-Abl enhances ganetespib recognition of wild-type hHsp90 (both α and β isoforms) (Figures 5A and 5B). The impact of phosphorylation of Y223-hAha1 on hHsp90 binding to ganetespib was further tested by transiently expressing hAha1 phospho-Y223F and Y223E mutants in c-Abl−/− MEF cells. Expression of the non-phospho Y223F mutant did not impact the Hsp90 binding to ganetespib, however, expression of hAha1-Y223E markedly reduced the Hsp90 affinity to ganetespib in c-Abl−/− MEF cells (Figures 5A and 5B). We further confirmed these data by showing c-Abl−/− MEF cells to be more sensitive to ganetespib, as evident by elevated levels of apoptotic markers at lower drug concentrations (Figure 5C). Although we cannot absolutely rule out the possibility that absence of c-Abl may indirectly sensitize the cells to Hsp90 inhibitors, the fact that overexpression of hAha1-Y223E in c-Abl−/− MEF cells reversed the drug sensitivity, reduces the likelihood of this hypothesis.

We next tested whether pharmacologic inhibition of c-Abl sensitizes cancer cells to Hsp90 inhibitors. The prostate cancer PC3 cell line was treated with different amounts of GNF-5 (a selective allosteric c–Abl inhibitor) for 24 hr (Zhang et al., 2010). We found that 5 μM GNF-5 is sufficient to inhibit c-Abl activity (assessed by level of Y-245 phosphorylation), (data not shown). We subsequently treated PC3 cells with 5.0 μM GNF-5 for 24 hr followed by 0–80 nM SNX2112, ganetespib, or PU-H71 (Hsp90 inhibitors) for an additional 24 hr (in the continued presence of GNF-5). As a control, we also treated PC3 cells with Hsp90 inhibitors, alone, for 24 hr. At the end of the incubation we quantified the percentage of apoptotic cells by flow cytometry (by measuring the increase of sub-G1 cellular DNA content Figures 5D, 5E, and S5). Compared to either GNF-5 or Hsp90 inhibitors alone, combined administration of both drugs had a synergistic effect on apoptosis (Figures 5D and 5E). These data were confirmed by assessing the abundance of cleaved caspase-3 and cleaved PARP, two cellular markers of apoptosis. Treatment as above with the combination of c-Abl inhibitor, GNF-5, (5 μM) and Hsp90 inhibitors (SNX2112 and ganetespib) significantly increased the level of apoptotic markers in PC3 cells, while similar incubation with the individual drugs was ineffective (Figure 5F).

Finally, we used two cell lines derived from human clear cell renal cell carcinoma (ccRCC), Caki-1 and 786-O. These cells were pretreated with 5 μM GNF-5 for 24 hr prior to addition of 40 and 80 nM ganetespib (Figure 5G). Pharmacologic inhibition of c-Abl also sensitizes these two ccRCC cell lines to ganetespib.

### Ex Vivo Inhibition of c-Abl Hypersensitizes Human RCC Tumors to Hsp90 Inhibitor Ganetespib

**Figure 6:**
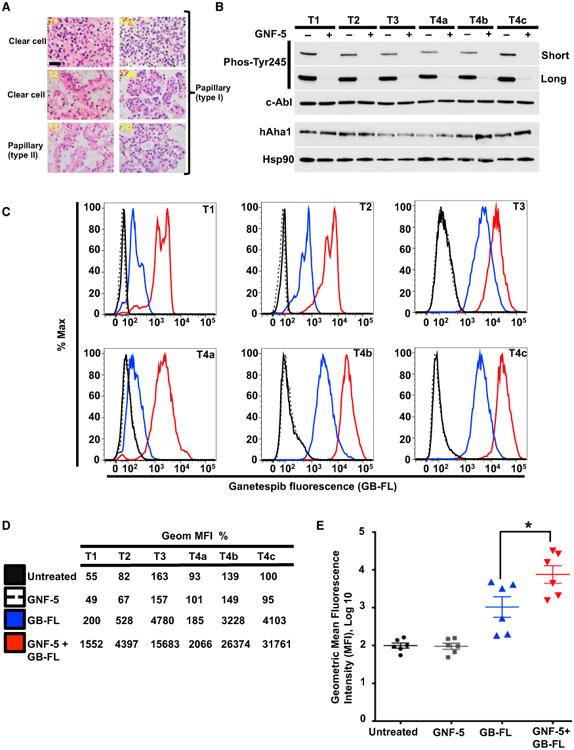
Pharmacologic Inhibition of c-Abl Increases Accumulation of Ganetespib in Human RCC Tumors from Patients (A) Human RCC tumors from patients were stained with hematoxylin and eosin (H&E). Patients 1 and 2 (P1 and P2) diagnosed with clear cell, Patient 3 (P3) with papillary type II, and Patient 4 (tumors 4a, 4b, and 4c) with papillary type I RCC are shown. The scale bar represents 20 μm. (B) Within 15 min of removal of tumors in (A) by radical nephrectomy, they were dissected into 3 mm^3^ pieces that were cultured in mediumcontaining 5 μM GNF-5 for 24 hr and then with 0.5 μM BODIPY FL-ganetespib (STA-12-9455) for an additional 6 hr. There were 10^7^ cells that were isolated from the tumors and the efficacy of c-Abl inhibition was confirmed by immunoblotting of the lysates with anti-phospho Y89-c-Abl monoclonalantibody. (C) Cells from (B) were stained with PI, fixed, and analyzed by flow cytometry. (D) Percentage (%) geometric mean fluorescence intensity (MFI) of the RCC cells from (B) treated with GNF-5 and/or FL-ganetespib. (E) Geometric mean fluorescence intensity (MFI) data from (C) were transformed to log_10_ values prior to statistical analysis. For each tumor treatment, a scatter dot plot with the mean and the error bars representing the SE of the mean (*p < 0.05) is shown.

ATP competitive inhibitors of Hsp90 such as ganetespib have the remarkable ability to accumulate in tumors, but not in normal tissues (Chan et al., 2008). Here, we used tumors from patients with RCC and observed whether inhibition of c-Abl increased their uptake of ganetespib. Based on histopathology, RCCs can be classified into several types. We used tumors from patients with ccRCC, papillary type I, and type II RCC (Figure 6A). Within 15 min of removal of tumors by radical nephrectomy, human RCC tumors were dissected into 3 mm^3^ pieces that were cultured in medium containing 5 μM GNF-5 for 24 hr and then with 0.5 μM boron-dipyrromethene (BODIPY) fluorophore-conjugated ganetespib (STA-12-9455), (FL-ganetespib) for an additional 6 hr.

Using an ex vivo approach, approximately 10^7^ cells were then isolated from these RCC solid tumors (Kedar et al., 1982). Western blot analysis showed that GNF-5 indeed inactivated c-Abl, and there also were relatively equal amounts of hAha1 and hHsp90 in these samples (Figure 6B). We next stained live cells with propidium iodide (PI) prior to fixation and analysis by flow cytometry. Our data show that RCC tumor cells were able to take up FL-ganetespib (Figure 6C). However, inhibition of c-Abl significantly increases the uptake of FL-ganetespib in these cells (Figures 6C–6E).

## Discussion

Co-chaperones are regulatory components of Hsp90, however, the underlying mechanisms of their regulation by PTMs are poorly understood. In this study, we show that hAha1 co-chaperone is phosphorylated on tyrosine residues. c-Abl was identified as the kinase that targets a conserved tyrosine-223 on hAha1 both in vitro and in vivo. This, in turn, promotes hAha1 interaction with hHsp90α. Mutation of Y223 to non-phosphorylatable phenylalanine (Y223F) disrupts hAha1 binding to hHsp90α and, subsequently, did not alter its ATPase activity. The phosphomimetic hAha1-Y223E mutant bound stronger to hHsp90α than the wild-type hAha1 and stimulated the hHsp90α ATPase activity by almost 8.5-fold relative to wild-type hAha1.

**Figure 7:**
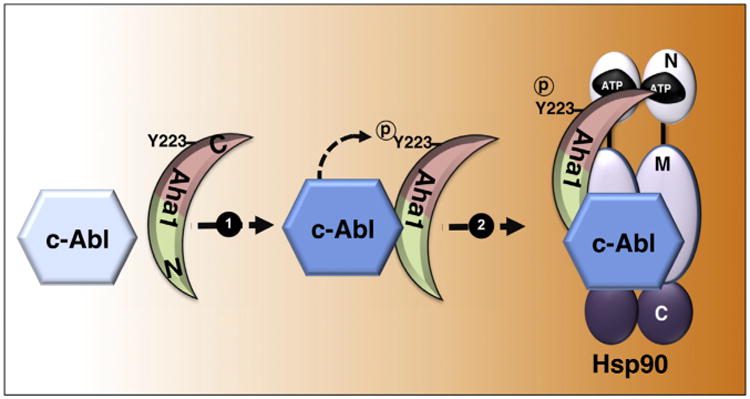
c-Abl Mediated Phosphorylation of the Co-chaperone hAha1-Y223 and Its Binding to Hsp90 c-Abl tyrosine kinase transiently binds and phosphorylates hAha1-Y223 (1). This, in turn, promotes its binding to Hsp90 and formation of hAha1-Hsp90-c-Abl complex (2).

Our previous work showed that bacterially expressed and purified hAha1 and hHsp90α, lacking any PTMs, interact and form a complex with each other, in vitro, and hAha1 also stimulates hHsp90α ATPase activity(Xu et al., 2012). PTMs of hHsp90, however, play a major role in its binding to the co-chaperones including hAha1 (Mollapour et al., 2011, 2014; Xu et al., 2012). Work by Buchner and co-workers has shown that yAha1 binding to yHsp90 is comprised of two steps. yAha1 N-domain (amino acids 1–156) makes initial contact with a motif in the yHsp90 middle-domain and this positions the yAha1 C-domain (amino acids 157–356) to interact with the yHsp90 N-domains (Li et al., 2013; Retzlaff et al., 2010). Tyrosine 223 is located in the C-domain of hAha1; thus, our data suggest that failure to establish contact with the hHsp90 N-domain significantly weakens overall stability of the complex, as previously suggested by Koulov et al. (2010). We therefore propose a regulatory paradigm where PTMs of Hsp90 (Mollapour et al., 2011, 2014; Xu et al., 2012) and (at least) tyrosine phosphorylation of hAha1-Y223 provide the signaling cues for association and complex formation of these two proteins (Figure7). While it cannot be formally excluded that enhanced interaction of phospho-hAha1 and Hsp90 may be facilitated by c-Abl kinase itself, we consider this very unlikely in view of the fact that phosphomimetic hAha1-Y223E mutant stimulated hHsp90α ATPase in vitro by 8.5-fold.

How does hAha1 tyrosine phosphorylation affect chaperoning of the clients? hAha1 plays a major role in chaperoning of v-Src, GR, and CFTR. Compared to the wild-type hAha1, the phosphomimetic Y223E mutant binds more strongly to hHsp90α and thus increases its ATPase activity, which appears to compromise the chaperoning of CFTR. Interestingly, the expression of the non-phosphorylatable hAha1-Y223F did not affect the folding of CFTR, providing further evidence that the Y223F mutant is unable to bind and stimulate Hsp90 ATPase activity. We have also shown that the phosphomimetic mutant Y223E elicited a stronger interaction in the hHsp90 in complex with kinase clients such as c-Abl, Raf-1, c-Src, Wee1, Ulk1, and Cdk4, as well as the co-chaperones PP5 and hHsp70. This could be the result of increased chaperone cycling that does not allow kinases and/or co-chaperones to dissociate from hHsp90. Using a yeast system to express the hAha1-Y223E in *yaha1*Δ mutant, we showed that the chaperoning of GR, Hsf1, and v-Src were significantly compromised. These observations may be the result of hyperactive chaperone cycle, therefore compromising the necessary dwell time between Hsp90 and the clients.

Phosphorylation of hAha1-Y223 causes ubiquitination and degradation of hAha1 in the proteasome. This indicates that the amount of phosphorylated Aha1 is tightly regulated. Additionally, the phosphomimetic Y223E tends to be retained in the cytoplasm compared to the wild-type hAha1 and non-phosphorylatable Y223F mutant localized both in the nucleus and cytoplasm. These data suggest that Aha1 may have additional cellular functions that are independent of binding and activating the Hsp90 ATPase activity. We explored this hypothesis, by performing a quantitative proteomic analysis with the phospho-hAha1 mutants. We observed hAha1 involvement in different cellular functions, especially interactions with components of the small (40S) and large (60S) subunits of the ribosome and ribosomal proteins such as elongation factors EF3 and EF4 (Figure S3B). This is consistent with previous work on hAha1 interactome (Sun et al., 2015) and also similar to the previously reported involvement of Hsp90 and co-chaperones Crp6/7 in ribosomal or pre-ribosomal functions (Kim et al., 2006; Schlatter et al., 2002; Tenge et al., 2014). Interestingly, we found that POM121 transmembrane nucleoporin dissociated from the phosphomimetic Y223E mutant (Figure S3B). Therefore, these data may partly explain the inability of hAha1-Y223E accumulation in the nucleus.

Hsp90 inhibitors are currently employed in clinical trials for treating cancer patients. Therefore, novel strategies to enhance their efficacy are actively sought. Previous data from Workman's research group has shown that downregulation of *hAHA1* results in increased tumor sensitivity to the first generation Hsp90 inhibitor, Tanespimycin (17-N-allylamino-17-demethoxygeldanamycin, 17-AAG) (Holmes et al., 2008). The authors also suggested a potential therapeutic strategy to increase the sensitivity of cancer cells to Hsp90 inhibitors by disrupting the hHsp90-hAha1 complex. Our data, here, provide further direct evidence that inhibition of c-Abl hypersensitizes prostate cancer and RCC cell lines to Hsp90 inhibitors, ganetespib and SNX2112. We have also shown that pharmacologic inhibition of c-Abl increases accumulation of ganetespib in cancer cells from patients with ccRCC, papillary type I, and type II RCC.

Our data also provide an explanation for previous findings on the synergistic effects of the combination of imatinib (STI571, Glivec, and Gleevec) plus 17-AAG on chronic myeloid leukemia (CML) cell lines (Radujkovic et al., 2005). Fusion of *bcr-abl* gene is associated with CML, and it has been shown that imatinib sensitizes CML cell lines to 17-AAG. Similar results were also observed with imatinib-resistant CML cell lines (Radujkovic et al., 2005). Based on our data, we speculate that imatinib mediated inhibition of Bcr-Abl, and possibly the endogenous c-Abl reduces the phosphorylation of hAha1-Y223, and therefore disrupts its association with Hsp90. This is turn enhances Hsp90 binding to its inhibitors.

## Experimental Procedures

### Statistical Analysis

The data presented are the representative or examples of three biological replicates unless it is specified. Data were analyzed with unpaired t test and oneway ANOVA analysis. Asterisks in figures indicate significant differences (* p < 0.05, ** p< 0.005, and *** p < 0.0005). The error bars represent the SD or SE for three independent experiments, unless it is indicated.

### Plasmids and Growth Media

Plasmids, a list of primers (Table S3), and media conditions for both yeast and mammalian cells are provided in the Supplemental Information.

### Protein Extraction, Co-immunoprecipitation, and Immunoblotting

Total protein extracts were prepared and analyzed by western blotting, as described previously (Mollapour et al., 2011). Detailed methods for protein extraction, precipitations, and detection by western blotting are presented in Supplemental Information.

### Assays for Hsp90 Client Activity

v-Src induction and activation were analyzed as described previously (Mollapour et al., 2011). Expressed v-Src protein was detected with EC10 mouse antibody (Millipore) and v-Src activity with 4G10 mouse anti-phosphotyrosine antibody (Millipore). GR assay was performed as described previously (Garabedian and Yamamoto, 1992), as was measurement of HSE-*LacZ* expression (Hjorth-Sørensen et al., 2001). *STE11*Δ*N* induction was analyzed as described previously (Flom et al., 2008; Louvion et al., 1998). Additional details are found in Supplemental Information.

### Immunofluorescence Staining and Microscopy

The procedures for immunofluorescence staining and confocal microscopy were described previously (Bourboulia et al., 2013). Detailed methodology is found in Supplemental Information.

### Flow Cytometry Analysis

PC3 cells treated with GNF-5, ganetespib, or SNX2112 were analyzed for SubG1 using PI. Uptake of fluorescently fluorescein isothiocyanate (FITC)-labeled ganetespib (STA-12-9455) (FL-ganetespib) by RCC cells was monitored by flow cytometry analysis. Detailed methodology is found in the Supplemental Information.

### hAha1 Comparative Interactome Analysis

Interactomes of hAha1-Y223E-FLAG and hAha1-Y223F-FLAG were analyzed as described previously (Truman et al., 2012). Briefly, hAha1-Y223E-FLAG or hAha1-Y223F-FLAG protein complexes were purified from 293T cells, trypsin digested, isotopically labeled, and subjected to LC-MS/MS. For each Hsp90 interactor, a log2 interaction change (Y223E/Y223F) was calculated after normalization to the bait protein (hAha1). The mass spectrometry proteomic data have been deposited to the ProteomeXchange Consortium via the PRIDE partner repository with the dataset identifier PXD001737. Detailed proteomic methods are provided in the Supplemental Information.

### Hsp90 ATPase Activity In Vivo

ATPase activity of hHsp90α isolated from prostate cancer PC3 cells, and its activation by hAha1 and phospho-Y223 mutants from HEK293 cells were measured as previously described (Kamal et al., 2003), with exceptions detailed in Supplemental Information.

### Ex Vivo Culture and Analysis of Human RCC Tumors

Tumor tissues of the patients with conventional RCC were obtained with written informed consent from the Department of Urology at SUNY Upstate Medical University. Patients had no history of hereditary VHL disease.

At the time of radical or partial nephrectomy, which was done with <15 min of renal ischemia, RCC tumors were dissected into 3–5 mm^3^ pieces and cultured in a presoaked gelatin sponge (Johnson and Johnson) in 24-well plates containing 2 ml RPMI-1640 with 10% FBS, antibiotic/antimycotic solution, with or without 5 μM GNF-5. Tissues were cultured at 37°Cfor24 hr followed by addition of 100 nM fluorescently labeled ganetespib (STA-12-9455), (FL-ganetespib) and further incubation at 37°C for 6 hr.

Using an ex vivo method as previously described (Kedar et al., 1982), approximately 10^7^ cells were isolated from the RCC solid tumors analysis by flow cytometry and western blot. All ex vivo experiments with the patients' samples were performed according to a protocol approved by the SUNY Upstate Medical University IRB.

## Highlights

- c-Abl phosphorylates the co-chaperone Aha1
- c-Abl phosphorylation of Aha1 tyrosine 223 promotes association with Hsp90
- Tyrosine phosphorylation of Aha1 leads to increased Hsp90 ATPase activity
- Pharmacological inhibition of c-Abl sensitizes cancer cells to Hsp90 inhibitors
